# Plasma Hormone and Metabolomics Identifies Metabolic Pathways Associated with Growth Rate of Dezhou Donkeys

**DOI:** 10.3390/ani15101435

**Published:** 2025-05-15

**Authors:** Liyuan Wang, Tong Li, Qiugang Ma, Honglei Qu, Changfa Wang, Wenqiang Liu, Wenqiong Chai

**Affiliations:** 1 Liaocheng Research Institute of Donkey High-Efficiency Breeding and Ecological Feeding, Liaocheng University, Liaocheng 252000, China; 2210190112@stu.lcu.edu.cn (L.W.); 2310190109@stu.lcu.edu.cn (T.L.); wangcf1967@163.com (C.W.); 2 State Key Laboratory of Animal Nutrition, College of Animal Science and Technology, China Agricultural University, Beijing 100193, China; maqiugang@cau.edu.cn (Q.M.); leihong_qu@163.com (H.Q.); 3 Gene-Marker Laboratory, Faculty of Agricultural and Life Science, Lincoln University, Lincoln 7647, Canterbury, New Zealand

**Keywords:** donkey, growth trait, hormones, metabolomics

## Abstract

Background: The growth traits of donkeys from the same farm under the same feeding conditions often vary. Methods: In this study, Plasma hormone level and LC–MS-based metabolomics was used to identify the metabolic pathways and the key metabolites associated with the growth rate of Dezhou donkeys. Results: The level of IGF-1 in the SG was significantly higher than that in the FG. The differentially abundant metabolites were related mainly to lipid metabolism, in which arachidonic acid metabolism, linoleic acid metabolism and steroid hormone biosynthesis played key roles. The main differentially abundant metabolites 2,3-dinor-8-iso-PGF2α, 11-DH-TXB2, 8(R)-HPETE, PGJ2, c9, t11-CLA, 12,13-DHOME, 9,10-DHOME, 9(10)-EpOME, 13-HPODE, DHEAS, testosterone, and corticosterone played important roles in metabolic homeostasis and affected the adaptation of donkeys to cold environments. Conclusions: The present study revealed that the growth rate of donkeys is mainly influenced by their adaptation to the environment, providing a more in-depth study on the relationship between plasma metabolomics and growth rate in donkeys.

## Simple Summary

The growth traits of donkeys from the same farm under the same feeding conditions often vary. The results showed that the level of IGF-1 in the SG was significantly higher than that in the FG. The differentially abundant metabolites were related mainly to lipid metabolism, in which arachidonic acid metabolism, linoleic acid metabolism and steroid hormone biosynthesis played key roles. The main differentially abundant metabolites 2,3-dinor-8-iso-PGF2α, 11-DH-TXB2, 8(R)-HPETE, PGJ2, c9, t11-CLA, 12,13-DHOME, 9,10-DHOME, 9(10)-EpOME, 13-HPODE, DHEAS, testosterone, and corticosterone played important roles in metabolic homeostasis and affected the adaptation of donkeys to cold environments.

## 1. Introduction

Donkeys were traditionally utilized as working animals in China. However, donkeys are no longer used as working animals due to technological development, and are instead used for meat, milk and skin production [1]. Compared with cattle and sheep, donkeys have a longer breeding cycle, which has made the stock decline in recent years, with only 1.46 million donkeys remaining by the end of 2023 [2]. Meanwhile, donkey breeding is relatively slow, with a low intraspecific homogeneity of traits. There are approximately 24 local donkey breeds in China. According to their physical appearance, they can be categorized into large-sized donkey breeds (Dezhou donkeys, Guanzhong donkeys and Jinnan donkeys), medium-sized breeds (Jiami donkeys, Biyang donkeys, and Qingyang donkeys) and small-sized breeds (Xinjiang donkeys, Liangzhou donkeys and Yunnan donkeys), with the heights above 130 cm, between 115 cm and 125 cm, and below 110 cm, respectively [1]. The Dezhou donkey is a large-sized breed. Adult male donkeys can weigh up to approximately 300 kg, the wither’s height and thoracic girth are usually up to 140 cm and 149 cm, respectively. Adult female donkeys can weigh up to 270 kg, and the wither’s height can be up to 135 cm and the thoracic girth can measure up to 145 cm [3,4]. According to a former investigation, the growth traits of donkeys vary under exactly the same feeding conditions within the same breed (unpublic data). Therefore, improving the growth traits of donkeys is important to improve their economic value.

The growth performance of animals is influenced by multiple factors such as breed, nutrition and the environment. Different breeds of animals exhibit different growth rates [5,6]. Environmental factors such as temperature, humidity and ventilation also affect the growth performance. In the meantime, the endocrine system can affect animal growth by regulating metabolism through hormones, such as growth hormone (GH), insulin-like growth factor (IGF-1), insulin (INS), thyroid hormones (THs), and androgens. Many studies have reported that the productive performance of animals is influenced by a variety of hormones. GH promotes lipolysis and regulates glucose metabolism as well as oxidative metabolism, and its promotion of muscle and bone development is accomplished primarily by stimulating the hepatic secretion of IGF-1 [7]. INS promotes myoglycogen synthesis and glucose uptake and utilization in skeletal muscle, hepatic glycogen synthesis and lipogenesis in the liver, inhibits lipolysis and promotes glucose uptake and lipogenesis in adipose tissue [8,9]. THs affect the body’s metabolic rate, energy balance, growth and development [10]. Androgens control the mechanical and energetic functions of muscle by regulating protein synthesis, glucose metabolism, and lipid metabolism, and they also regulate the distribution of body fat through adipose tissue [11]. It has been reported that the secretion level of IGF-1 is phenotypically correlated with the live weight and growth rate of livestock [12]. Broilers and rabbits with high growth performance presented high levels of GH, INS, IGF-1 and THs [13,14].

Metabolomics involves the analysis of small molecules (metabolites) in biological cells, tissues, and body fluids to detect subtle alterations in biological pathways and provide insight into the mechanisms. The metabolome has been used to explore metabolic processes and key metabolites associated with animal species, growth and development, and stress responses [15]. Therefore, this study has selected Dezhou donkeys with different growth rates and plasma metabolomics was performed to identify and analyze the metabolic pathways and the marker metabolites associated with the growth rate of Dezhou donkeys, which will provide a basis for the future improvement of donkey growth performance.

## 2. Materials and Methods

### 2.1. Animals and Sample Collection

The experimental procedures were reviewed and approved by the Liaocheng University Animal Care and Ethics Committee (No. 2023042602). A total of 16 9-month-old male Dezhou donkeys (initial weight of 152.66 ± 19.49 kg) were randomly selected, and body size and weight were measured at Yucheng Huimin Agricultural Science and Technology Company (Dezhou, China). All donkeys were kept in the same enclosure with roof and fences; the feeding conditions were exactly same (from early July 2023 to December 2023) and weighed once a month. In early December 2023, the body size was measured, and venous blood was collected. Blood samples were collected using 10 mL blood collection tubes (Kangweishi Medical Technology Co., Ltd., Shijiazhuang, China) with EDTA anticoagulant and left at room temperature for 2 h, followed by centrifugation at 3000 rpm for 10 min. The plasma samples were harvested and stored at −80 °C.

### 2.2. Determination of Growth Performance

Body size indicators, including body weight (BW), body slant length (BSL), body height (BH), chest circumference (CC), chest width (CW), chest depth (CD), rump height (RH), rump length (RL), rump width (RW) and cannon bone circumference (BC). The average daily weight gain (ADG) was calculated as the difference between the final weight and initial weight divided by the number of days. All donkeys were divided into two groups, categorized as fast-grown group (FG) and slow-grown group (SG) based on the ADG. Ten donkeys were finally selected randomly from each group for the follow-up analyses. Hormone levels were detected, and metabolomics analyses were performed.

### 2.3. Hormone Level Measurement

Donkey growth hormone (GH), testosterone (T), thyroxine (T4), and insulin (INS) levels were measured via radioimmunoassay. Insulin-like growth factor-1 (IGF-1) was measured using enzyme-linked immunosorbent assay (ELISA). Measurements were performed according to the instructions of individual hormone radioimmunoassay kits (Beijing North Institute of Biotechnology Co., Ltd., Beijing, China) and horse IGF-1 ELISA Kit (ShangHai Xinfan Biotechnology Co., Ltd., Shanghai, China).

### 2.4. Metabolomic Analysis

LC–MS untargeted metabolomics determination was performed at Biomarker Technologies Co., Ltd. (Beijing, China).

#### 2.4.1. Metabolite Extraction

A total of 500 μL of methanol/acetonitrile (*v*/*v*: 1:1) containing an internal standard (20 mg/L) was added to 100 μL of each plasma sample, and the mixture was vortexed for 30 s. The mixture was sonicated in an ice-water bath for 10 min and placed at −20 °C for 1 h. Then centrifuged at 12,000 rpm and 4 °C for 15 min, and 500 μL of the supernatant was aspirated for vacuum drying. Acetonitrile/water (*v*/*v*: 1:1, 160 μL) was used to redissolve the dried metabolite, which was vortexed for 30 s, followed by sonication in an ice-water bath for 10 min. After centrifugation at 12,000 rpm for 15 min (4 °C), 120 μL of the supernatant was transferred to a 2 mL injection vial. The QC sample was prepared by mixing 10 μL of each sample.

#### 2.4.2. LC–MS/MS Detection

The LC/MS system for metabolomics analysis was composed of a Waters Acquity I-Class PLUS ultrahigh-performance liquid (Waters Corporation, Milford, MA, USA) coupled with a Waters Xevo G2-XS QTof high-resolution mass spectrometer (Waters Corporation, Milford, MA, USA). The column used was purchased from a Waters ACQUITY UPLC HSS T3 column (1.8 μm, 2.1 mm × 100 mm; Waters Corporation, Milford, MA, USA). The liquid conditions were as follows: mobile phase A, 0.1% formic acid aqueous solution; mobile phase B, 0.1% formic acid acetonitrile; 0–0.25 min, A, 98%; 0.25–10 min, A, 98–2%; 10–13 min, A, 2%; 13–13.1 min, A, 2–98%; and 13.1–15 min, A, 98%. The parameters of the ESI ion source were as follows: capillary voltage: 2000 V (positive ion mode) or −1500 V (negative ion mode); cone voltage: 30 V; ion source temperature: 150 °C; desolvent gas temperature: 500 °C; backflush gas flow rate: 50 L/h; and desolventizing gas flow rate: 800 L/h.

#### 2.4.3. Data Processing

Raw data collected using MassLynx V4.2 were processed by Progenesis QI v3.0 software (WatersCorporation, Milford, CT, USA) for peak extraction, peak alignment and identification. After normalizing the original peak area information with the total peak area, follow-up analysis was performed. To display group separation and identify significantly changed metabolites, we applied the supervised orthogonal projection to latent structure discrimination analysis (OPLS-DA). To verify the reliability of the model, 200 permutation tests were performed. The screening criteria were FC ≥ 2 or ≤0.5, *p*-value < 0.05 and VIP > 1. The identified compounds were searched for classification and pathway enrichment in the Human Metabolome Database (HMDB) and Kyoto Encyclopedia of Genes and Genomes (KEGG) databases. The KEGG pathway enrichment significance was calculated using hypergeometric distribution test.

### 2.5. Statistical Analysis

SPSS 26.0 software (IBM, Armonk, NY, USA) was used, and all the data are expressed as the mean ± standard error (SE). Independent-sample *t*-tests were conducted to determine significant differences between means. The significance level was *p* < 0.05.

## 3. Results

### 3.1. Determination of Growth Performance of Donkeys

**Figure 1:**
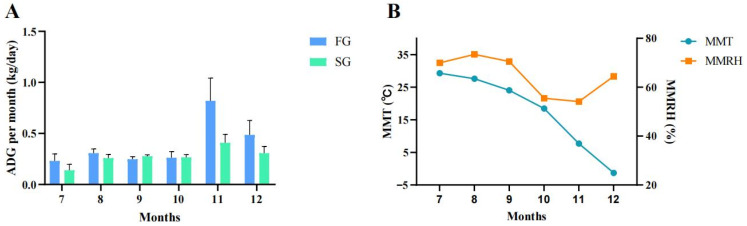
The growth rate per month for two groups (*n* = 5). (**A**) ADG for each month between FG and SG. (**B**) Mean temperatures and humidity of each month. FG: fast group; SG: slow group; ADG: average daily weight gain; MMT: mean monthly temperature; MMRH: mean monthly relative humidity.

In this study, initial BW (IBW) and body size were measured at 9 months of age, and final BW (FBW) and body size were measured at 14 months of age. As shown in Table 1, the final BSL, CC, CW, RW and BC were significantly higher than those of the initial values (*p* < 0.05). The ADG in the FG was significantly higher than SG (*p* < 0.05) (Table 2). The ADG for each month of the two groups was calculated (Figure 1). As shown in Figure 1A, from July to December, the ADG in the FG was higher than that in the SG, except for September and October, when the ADG in the FG was slightly lower than that in the SG, but there was a dramatic difference in November. As shown in Figure 1B, the mean monthly temperature (MMT) decreased gradually from July (29.3 °C) to December (−1.3 °C), with a faster rate of decrease in November and December. The mean monthly relative humidity (MMRH) was lower in October (55%) and November (54%) than in the other months (64–73%).

### 3.2. Hormone Levels of Donkeys

**Figure 2:**
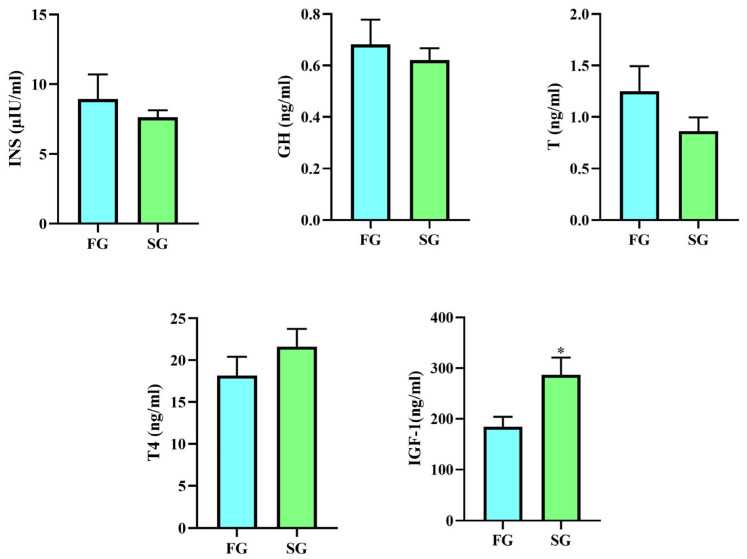
Growth-related hormone levels in donkeys (*n* = 5). FG: fast group; SG: slow group; INS: insulin; GH: growth hormone; T: testosterone; T4: thyroxine; IGF-1: Insulin-like growth factor 1. * represents *p* < 0.05.

Hormones play important roles in animal growth. In the present study, the levels of growth hormone (GH), testosterone (T), thyroxine (T4), insulin (INS) and insulin-like growth factor-1 (IGF-1) were determined (Figure 2). The concentration of IGF-1 in the SG was significantly higher than that in the FG. There were no significant differences in GH, T, T4, or INS between the two groups. The concentrations of INS and GH in the FG were higher than those in the SG, and the level of T4 was lower than that in the SG.

### 3.3. Metabolomic Profiling of Plasma Samples

#### 3.3.1. Analysis of Plasma Differentially Abundant Metabolites

**Figure 3:**
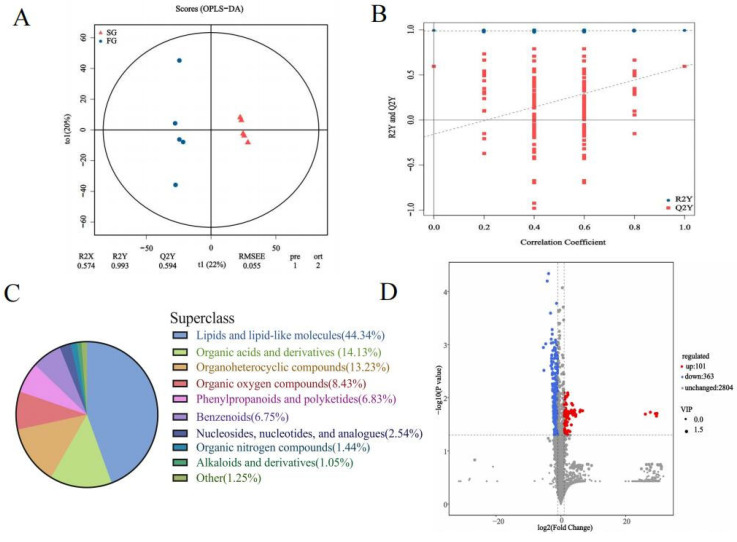
(**A**) OPLS-DA model plot of the identified metabolites (*n* = 5). (**B**) Validation of the OPLS-DA model via permutation testing (200 iterations). (**C**) Sector diagram of metabolite classification and proportions. (**D**) Volcano plots showing differentially abundant metabolites.

The chromatographic peaks in the QC samples were almost all consistent in positive and negative modes, indicating that the detection process had good stability and reproducibility. As shown in the score plots of OPLS-DA (Figure 3A), the FG was separated from the SG, indicating significant differences in the plasma metabolic profile between the two groups. R2X, R2Y and Q2 were 0.574, 0.993 and 0.594, respectively. The results of the 200 permutation tests showed that the slopes of R2Y and Q2Y were moderate and that the intercept of Q2Y was negative. (Figure 3B). These results indicated that the OPLS-DA model had good predictive ability and was not overfitted. A total of 3268 named metabolites were identified in the donkey plasma, of which 2562 had chemical classification information. Most of the metabolites were classified as lipids and lipid-like molecules (44.34%), followed by organic acids and derivatives (14.13%), organoheterocyclic compounds (13.23%), and organic oxygen compounds (8.43%) (Figure 3C). Differential analysis of the identified plasma metabolites revealed that 363 metabolites were downregulated and 101 metabolites were upregulated in the FG compared with the SG (Figure 3D). The top 10 significantly upregulated and top 10 significantly downregulated differentially abundant metabolites are shown in Table 3. The FC, *p*-value, VIP and annotation details for 464 differentially abundant metabolites can be seen in Supplementary File S1.

#### 3.3.2. Functional Enrichment Analysis of Differentially Abundant Metabolites

**Figure 4:**
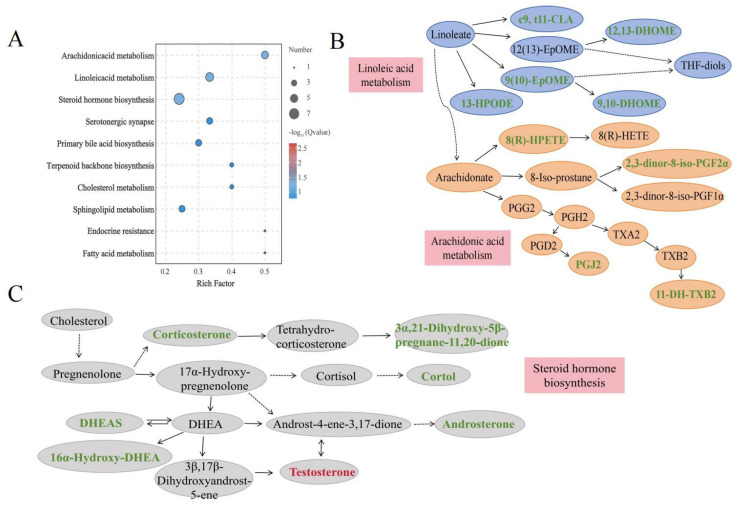
KEGG enrichment dot plot of plasma differentially abundant metabolites. (**A**) KEGG enrichment dot plot. (**B**) Pathway diagrams for linoleic acid metabolism and arachidonic acid metabolism, (**C**) steroid hormone biosynthesis. Green-labeled metabolites indicate downregulation and red-labeled metabolites indicate upregulation in the FG group. CLA: conjugated linoleic acid; 13-HPODE: 13-hydroperoxyoctadecadienoic acid; DHOME: dihydroxyoctadecenoic acid; EPOME: epoxyoctadecenoic acid; HPETE: hydroxyperoxyeicosatetraenoic acid; HETE: hydroxyeicosatetraenoic acid; 11-DH-TXB2: 11-dehydro-thromboxane B2; PG: prostaglandin; DHEA: dehydroepiandrosterone; DHEAS: dehydroepiandrosterone-sulfate.

To understand the metabolic pathways associated with growth rate, metabolic pathway enrichment analysis based on the KEGG database was performed (Figure 4A). The pathway enrichment analysis revealed that the differentially abundant metabolites were enriched mainly in lipid metabolism pathways, including linoleic acid metabolism, arachidonic acid metabolism and steroid hormone biosynthesis, all of which were identified to be the key pathways involved in animal growth (Table 4). The pathway diagrams for linoleic acid metabolism, arachidonic acid metabolism, and steroid hormone biosynthesis can be seen in Figure 4B,C. The differentially abundant metabolites 2,3-dinor-8-iso-PGF2α, 11-DH-TXB2, 8(R)-HPETE, PGJ2, c9, t11-CLA, 12,13-DHOME, 9,10-DHOME, 9(10)-EpOME, 13-HPODE, DHEAS, testosterone, and corticosterone were involved in metabolic homeostasis.

## 4. Discussion

Productive indicators such as body weight and body size can be used to visualize an animal’s growth and nutritional status [16]. Compared with other large donkey breeds, such as Jiangyue and Guanzhong donkeys, male Dezhou donkeys were reported to have the highest body weight before 12 months of age but lower body weights than other breeds at 18 and 24 months of age, suggesting that Dezhou donkeys have a shorter growth and developmental peak than other breeds [17]. Donkeys from the same farm presented different growth rates, which were reflected by their ADG. However, there are no studies related to the ADG of donkeys. Animal growth is influenced by metabolic processes, such as glucose metabolism, lipid metabolism, and protein metabolism, which are regulated by multiple factors. Hormones such as GH, IGF-1, INS, T and TH can influence animal growth processes by regulating various metabolic processes. The results revealed that IGF-1 was significantly different between the two groups. IGF-1 is generally recognized as a positive regulator of animal growth. However, IGF-1 affects animal growth through autocrine and paracrine mechanisms rather than endocrine secretion, and a large decrease in blood IGF-1 concentration due to hepatic IGF-1 gene deletion has little effect on normal postnatal growth and development [18,19]. This explains the higher plasma IGF-1 concentration in the SG group, but not the faster growth rate. The present study spanned from summer (temperature approx. 28 °C, humidity approx. 70%) to winter (temperature approx. −1 °C, humidity approx. 64%), the decreases in temperature and humidity may have also affected donkey growth. When animals are exposed to cold environments, maintenance energy metabolism increases and production energy metabolism decreases to adapt to the environment, resulting in a decrease in productive performance and immunocompetence [20,21]. The adaptation to the environment is regulated by hormones. Animals subjected to stimuli first through the locus coeruleus-noradrenergic neuron–sympathetic adrenal medulla axis (SAM axis) release a variety of hormones for a timely response. Subsequently, this activates the hypothalamo–pituitary–adrenal axis (HPA axis) to release glucocorticoids to regulate cold stress. Activation of the HPA axis may also cause an increase in DHEAS secretion [22]. The hypothalamic–pituitary –thyroid axis (HPT axis) increases the metabolic rate primarily through the release of triiodothyronine (T3) and T4, which results in the production of more calories [23]. In this study, the SG presented higher levels of IGF-1, DHEAS and corticosterone, and lower T levels. It was reported that macrophage-derived IGF-1 in adipose tissue plays an important role in maintaining adipose tissue mass during the response to cold stress [24]. DHEAS, which is the precursor of T, is the predominant form of DHEA in circulation [25]. The level of T directly affects skeletal development during puberty and can indirectly affect growth by influencing growth hormone levels [26]. However, cold stimulation can lead to a decrease in testosterone production [27]. The hormones with significant differences between the FG and SG groups were related mainly to cold stress, and the HPA axis response was more drastic in the SG group, which indicated that its adaptation to the cold environment was lower than that of the FG group.

Plasma metabolites were reported to be correlated with body weight and growth rate in animals [28]. In cold environments, nutrients oxidize and break down to release more heat, which produces free radicals and causes oxidative damage to the body [29]. Prolonged exposure of animals to cold stress results in inflammatory damage [30]. In the present study, linoleic acid metabolism and arachidonic acid metabolism play important roles in inflammation, immune response and regulation of lipid metabolism. 8-iso-PGF2α, 2,3-dinor-8-iso-PGF2α and 11-DH-TXB2 are biomarkers of oxidative damage and platelet activation in the body [31]. In addition, 2,3-dinor-8-iso PGF2α is a metabolite of 8-iso PGF2α and is chemically more stable. There has been a report that showed that 12-HPETE and 15-HPETE inhibit cell proliferation, induce apoptosis, and are associated with oxidative stress and inflammatory responses [32]. 13-HPODE has been shown to have pro-inflammatory properties, and EpOMEs and DHOMEs increase oxidative stress [33]. PGJ2 was reported to mediate anti-inflammatory effects, and CLA increases the levels of oxidative stress markers [malondialdehyde (MDA) and 8-iso-PGF2α] [34]. Subsequent studies revealed that the CLA with anti-obesity effects is t10, c12-CLA, whereas c9, t11-CLA plays more important roles in anti-inflammatory and immunomodulation [35]. Additionally, DHEA and DHEAS have anti-inflammatory, antioxidant and immunomodulatory properties [36]. In the present study, the metabolites of AA [2,3-dinor-8-iso-PGF2α, 11-DH-TXB2, 8(R)-HPETE] and the metabolites of LA [13-HPODE, 9(10)-EpOME, 12,13-DHOME, and 9,10-DHOME] were upregulated in the SG, indicating cold stress-induced inflammatory responses and oxidative damage in the donkeys of the SG. Moreover, the c9, t11-CLA, PGJ2, and DHEAS levels were higher in the SG, which may be the result of the defense of the organism against cold stress by increasing their levels. In the present study, most differentially abundant metabolites and hormones with higher levels were associated with oxidative stress and inflammatory responses, suggesting that donkeys in the SG had lower immunocompetence and higher oxidative damage under cold stress. A previous study reported that the addition of windbreaks in cold environments improved donkey growth performance [37]. Therefore, the performance of donkeys can be improved by adding cold protection during their breeding.

The differential plasma metabolites of the donkeys in this study were related mainly to lipid metabolism, suggesting that their fast and slow growth rates are strongly influenced by the level of lipid metabolism. Conjugated linoleic acid (CLA) is a family of isomers of linoleic acid, of which c9, t11-CLA and t10, c12-CLA were shown to be biologically active in a variety of ways. In both subcutaneous fat and intramuscular fat, a linear response of c9,t11-CLA and t10,c12-CLA to dietary CLA has been reported [38]. Dietary CLA reduces subcutaneous fat deposition primarily by promoting energy metabolism, inhibiting lipogenesis, and promoting lipolysis, but increases IMF deposition by promoting lipogenesis [39,40]. Moreover, the regulation of fat deposition by CLA is realized through the expression of related genes, such as the fatty acid binding protein-encoding gene and peroxisome proliferator-activated receptor (PPAR) [41]. In addition, the linoleic acid metabolites 12,13-DHOME and 9,10-DHOME, 12(13)-EpOME, 9(10)-EpOME, and 13-HPODE could also function as ligands for PPAR to regulate adipocyte differentiation and lipid metabolism [42]. The upregulation of c9, t11-CLA, 12,13-DHOME, 9,10-DHOME, 9(10)-EpOME, and 13-HPODE in the SG suggested that these donkeys regulate more lipid metabolism, which may be related to their lower growth performance.

## 5. Conclusions

The present study provides a deeper insight into the relationships among hormone levels, plasma metabolomics and growth rates in donkeys. The results showed that the level of IGF-1 in the SG was significantly higher than that in the FG. The fast and slow growth rates were influenced mainly by lipid metabolism, in which arachidonic acid metabolism, linoleic acid metabolism, and steroid hormone biosynthesis played key roles. In addition, the differentially abundant metabolites 2,3-dinor-8-iso-PGF2α, 11-DH-TXB2, 8(R)-HPETE, PGJ2, c9, t11-CLA, 12,13-DHOME, 9,10-DHOME, 9(10)-EpOME, 13-HPODE, DHEAS, testosterone, and corticosterone play important roles in metabolic homeostasis and affect donkey adaptability to cold environments. The slow growth rate of donkeys may be due to their weak adaptability to the cold environment and cold stress. Therefore, the growth performance of Dezhou donkeys can be improved in the future by the addition of cold protection facilities.

## Figures and Tables

**Table 1:** Body size measurement of FG and SG.

Traits	FG-Initial	SG-Initial	FG-Final	SG-Final
BH (cm)	120.6 ± 2.5	119.5 ± 2.5	127.0 ± 2.4	124.8 ± 2.7
BSL (cm)	111.0 ± 2.7 b	110.2 ± 0.8 b	123.4 ± 1.9 a	122 ± 2.0 a
CD (cm)	45.7 ± 0.9	46.3 ± 1.2	48 ± 0.8	48.8 ± 1.0
CC (cm)	119.8 ± 1.3 b	120.4 ± 2.0 b	130.3 ± 1.8 a	129.6 ± 2.0 a
CW (cm)	27.2 ± 0.4 b	27.6 ± 0.4 b	31.2 ± 1.0 a	31.2 ± 0.3 a
RH (cm)	122.9 ± 2.8	120.6 ± 2.8	131.1 ± 2.1	128.7 ± 3.0
RL (cm)	36.4 ± 0.9 b	36.2 ± 0.6 b	39.4 ± 1.2 ab	40 ± 0.8 a
RW (cm)	31.8 ± 0.3 b	31.4 ± 0.8 b	34.9 ± 0.5 a	34.7 ± 0.4 a
BC (cm)	14.4 ± 0.3 b	14.6 ± 0.4 b	16.7 ± 0.5 a	16.3 ± 0.2 a

Note: *n* = 5. FG: fast group; SG: slow group; BW: body weight; BSL: body slant length; BH: body height; CC: chest circumference; CW: chest width; CD: chest depth; RH: rump height; RL: rump length; RW: rump width; BC: cannon bone circumference. Values with different letters in the same row indicate significant differences (*p* < 0.05), and those with no letter or the same letter in the same row indicate non-significant differences (*p* > 0.05).

**Table 2:** Body weight of FG and SG.

Items	FG	SG	*p*-Value
IBW (kg)	159.36 ± 8.32	166.35 ± 3.61	0.463
FBW (kg)	212.80 ± 10.43	203.60 ± 7.32	0.491
ADG (kg/day)	0.35 ± 0.02 a	0.24 ± 0.03 b	0.024

Note: *n* = 5. FG: fast group; SG: slow group; IBW: initial body weight; FBW: final body weight; ADG: average daily weight gain. Values with different letters in the same row indicate significant differences (*p* < 0.05), and those with no letter in the same row indicate non-significant differences (*p* > 0.05).

**Table 3:** The top 10 significantly upregulated and top 10 significantly downregulated differentially abundant metabolites.

Differential Abundant Metabolites	log_2_FC	*p*-Value	VIP
(3b,6b,8a,12a)-8,12-Epoxy-7(11)-eremophilene-6,8,12-trimethoxy-3-ol	29.68	0.020	1.70
PA (14:0/PGJ2)	29.52	0.022	1.70
Bacoside A	29.49	0.021	1.72
solasodine 3-O-beta-D-glucopyranoside	29.18	0.020	1.75
Tryptophyl-Valine	27.65	0.019	1.72
Prizidilol	26.09	0.020	1.70
Heteroxanthin	6.71	0.018	1.74
DG (8:0/PGE1/0:0)	6.01	0.017	1.73
Annomuricin A	5.87	0.018	1.76
Dihydromycoplanecin A	4.60	0.021	1.69
1-Stearoyl-2-hydroxy-sn-glycero-3-phosphocholine	−5.40	0.001	2.01
Retinyl palmitate	−5.24	0.003	1.96
Isobutyl 10-undecenoate	−4.70	0.001	1.99
(±)-(E)-3-Methyl-4-decen-1-yl acetate	−4.22	0.000	2.04
Hexosylsphingosine	−3.80	0.000	2.03
DG (8:0/0:0/17:0)	−3.20	0.000	1.97
apicidin	−3.07	0.003	1.88
Neomycin B	−2.98	0.001	1.89
12-Hydroxy-12-octadecanoylcarnitine	−2.96	0.001	1.93
Glycocholate	−2.91	0.002	1.96

**Table 4:** The significantly enriched pathways and differentially abundant metabolites related to donkey growth in KEGG pathways.

KEGG Pathway	Differentially Abundant Metabolites
Linoleic acid metabolism	c9,t11-conjugated linoleic acid (c9, t11-CLA), dihydroxyoctadecenoic acids (9,10-DHOME, 12,13-DHOME), epoxyoctadecenoic acids (9(10)-EpOME), 13-hydroperoxyoctadecadienoic acid (13(S)-HPODE)
Arachidonic acid metabolism	8(R)-hydroxyperoxyeicosatetraenoic acid (8(R)-HPETE), prostaglandin J2 (PGJ2), 2,3-dinor-8-iso-PGF2α, 11-dehydro-thromboxane B2 (11-DH-TXB2)
Steroid hormone biosynthesis	corticosterone, cortol, dehydroepiandrosterone-sulfate (DHEAS), androsterone, testosterone
Primary bile acid biosynthesis	taurocholic acid, glycocholate, 3α,7α,26-trihydroxy-5β-cholestane
Sphingolipid metabolism	galabiosylceramide, sphingosylphosphorylcholine, 3-O-Sulfogalactosylceramide

## Data Availability

The original contributions presented in the study are included in the article/Supplementary Materials, and further inquiries can be directed to the corresponding authors.

## Supplementary Materials

The following supporting information can be downloaded at: https://www.mdpi.com/article/10.3390/ani15101435/s1, Supplementary File S1: The FC, *p*-value, VIP and annotation details for 464 differentially abundant metabolites.
