# Supplementary material for: Plasma Hormone and Metabolomics Identifies Metabolic Pathways Associated with Growth Rate of Dezhou Donkeys
Source: Animals (Basel). 2025 May 15;15(10):1435. doi: 10.3390/ani15101435 (PMC12108408; doi:10.3390/ani15101435)
Supplement: Supplementary file 1 [file animals-15-01435-s001.zip › animals-3634117-supplementary.pdf]

Table S1. Listing all 464 differentially abundant metabolites

**Metabolites name**	**Fold change**	**log2FC**	***P* value**	**VIP**	**regulated**
3',4'-Dihydrorhodovibrin	0.33	-1.58	0.007	1.68	down
Medrogestone	0.45	-1.15	0.032	1.54	down
Annonin XIV	0.36	-1.49	0.026	1.58	down
(3beta,19alpha)-3,19,23,24-Tetrahydroxy-12-oleanen-28-oic acid	0.22	-2.21	0.007	1.73	down
Cer(d16:1/22:6(5Z,8E,10Z,13Z,15E,19Z)-2OH(7S,17S))	0.34	-1.55	0.023	1.64	down
PG(18:0/PGF1alpha)	0.28	-1.84	0.021	1.66	down
Spiramycin 1	0.21	-2.28	0.012	1.67	down
Volicitin	0.45	-1.15	0.017	1.61	down
L-Glutamyl 5-phosphate	2.04	1.03	0.026	1.50	up
2-(1-Ethoxyethoxy)propanoic acid	0.41	-1.28	0.034	1.42	down
9-(3-Methyl-5-propylfuran-2-yl)nonanoylcarnitine	2.92	1.55	0.021	1.65	up
Diaveridine	5.69	2.51	0.019	1.67	up
3-Hydroxydodeca-6,9-dienoylcarnitine	2.81	1.49	0.016	1.73	up
Merodesmosine	2.34	1.23	0.012	1.75	up
(2S)-N-[(2S)-1-[Acetyl-[(2S)-1-oxohexan-2-yl]amino]-4-methyl-1-oxopentan-2-yl]-2-amino-4-methylpentanamide	4.94	2.30	0.018	1.71	up
PG(18:0/PGF2alpha)	2.70	1.43	0.021	1.63	up
Schleicherastatin 5	3.19	1.67	0.029	1.57	up
Furanofukinin	2.15	1.10	0.017	1.67	up
1-(1-Hydroxy-2,2,6,6-tetramethylpiperidin-4-yl)-1h-pyrrole-2,5-dione	2.71	1.44	0.026	1.56	up
PE(22:0/20:4(6Z,8E,10E,14Z)-2OH(5S,12R))	2.78	1.48	0.009	1.73	up
Lc3Cer	0.25	-2.00	0.040	1.41	down
PGP(i-20:0/20:3(5Z,8Z,11Z)-O(14R,15S))	0.22	-2.20	0.019	1.56	down
Avermectin A1b monosaccharide	0.38	-1.39	0.022	1.58	down
(6E)-Undec-6-enoylcarnitine	2.47	1.31	0.013	1.68	up
Meproscillarin	0.44	-1.19	0.024	1.57	down
Galabiosylceramide (d18:1/24:1(15Z))	2.00	1.00	0.008	1.74	up
Abemaciclib	3.28	1.71	0.030	1.60	up
PGP(i-22:0/PGF1alpha)	13.85	3.79	0.021	1.69	up
Hordatine B	23.71	4.57	0.022	1.67	up
PA(21:0/14:0)	2.01	1.01	0.032	1.49	up
CDP-N-dimethylethanolamine	2.02	1.01	0.010	1.62	up
1alpha-Hydroxycorticosterone	0.27	-1.91	0.047	1.36	down
PG(a-13:0/PGF2alpha)	0.29	-1.80	0.048	1.36	down
Undec-5-enedioylcarnitine	0.32	-1.66	0.049	1.34	down
Bopindolol	0.30	-1.72	0.002	1.83	down
4-Butyl-5-ethylthiazole	0.38	-1.39	0.013	1.60	down

Talbutal	0.46	-1.12	0.017	1.62	down
DG(2:0/6 keto-PGF1alpha/0:0)	0.26	-1.94	0.040	1.48	down
Taurocholic acid	0.25	-1.99	0.028	1.53	down
N-[Diaminophosphoryloxy-[di(propan-2-yl)amino]phosphoryl]-N-propan-2-ylpropan-2-amine	0.49	-1.03	0.019	1.66	down
2-Hydroxy-2,6,6-trimethylcyclohexanone	0.24	-2.05	0.026	1.53	down
(1-Methyl-2,3-dihydropyridin-6-ylidene)hydrazine	0.31	-1.71	0.014	1.66	down
Resolvin D2	0.33	-1.62	0.002	1.84	down
Dehydroepiandrosterone sulfate	0.43	-1.23	0.010	1.67	down
9,10-Epoxyoctadecenoic acid	0.40	-1.31	0.008	1.76	down
12-hydroxyheptadecanoic acid	0.26	-1.96	0.018	1.69	down
1-Nitrohexane	0.44	-1.19	0.020	1.69	down
12-oxo-PDA	0.43	-1.22	0.020	1.52	down
(S)-10,16-Dihydroxyhexadecanoic acid	0.29	-1.77	0.009	1.64	down
(3R,5Z)-5-Octene-1,3-diol	0.20	-2.35	0.014	1.76	down
(9Z,11E)-(13S)-13-Hydroperoxyoctadeca-9,11-dienoic acid	0.45	-1.16	0.004	1.88	down
19-Hydroxydeoxycorticosterone	0.20	-2.32	0.012	1.72	down
11-Dehydro-thromboxane B2	0.25	-2.00	0.020	1.65	down
PG(i-14:0/PGF1alpha)	0.28	-1.84	0.023	1.64	down
PA(22:4(7Z,10Z,13Z,16Z)/6 keto-PGF1alpha)	0.30	-1.72	0.024	1.62	down
PGP(i-22:0/18:1(12Z)-O(9S,10R))	0.33	-1.58	0.026	1.60	down
2,3-Dinor-11b-PGF2a	0.45	-1.16	0.041	1.42	down
Ajulemic acid	0.42	-1.26	0.023	1.62	down
9,10-Epoxyoctadecanoic acid	0.39	-1.35	0.021	1.69	down
8(R)-HPETE	0.40	-1.31	0.022	1.68	down
DG(15:0/20:4(7E,9E,11Z,13E)-3OH(5S,6R,15S)/0:0)	0.24	-2.08	0.040	1.38	down
(±)9-HODE	0.25	-1.98	0.018	1.70	down
Ganglioside GM3 (d18:1/16:0)	2.90	1.53	0.020	1.71	up
4-Hydroxynonenal	0.43	-1.20	0.025	1.60	down
N-Lauroyl Glutamine	0.48	-1.06	0.023	1.64	down
9-Oxo-nonanoic acid	0.26	-1.95	0.043	1.49	down
(R)-6-(4-((4-Ethylpiperazin-1-yl)methyl)phenyl)-N-(1-phenylethyl)-7H-pyrrolo[2,3-d]pyrimidin-4-amine	0.29	-1.78	0.018	1.69	down
(±)-(E)-13-Hydroxy-10-oxo-11-octadecenoic acid	0.45	-1.16	0.033	1.52	down
(±)12,13-DiHOME	0.50	-1.01	0.021	1.68	down
DG(15:0/PGE2/0:0)	0.22	-2.18	0.016	1.61	down
PA(P-16:0/PGE1)	0.21	-2.24	0.018	1.68	down
PE-NMe2(14:0/20:1(11Z))	0.41	-1.28	0.007	1.69	down
6,15-Diketo,13,14-dihydro-PGF1a	0.23	-2.14	0.024	1.64	down
PGP(i-22:0/18:1(9Z)-O(12,13))	0.30	-1.76	0.029	1.59	down

Didemnin C	0.28	-1.82	0.048	1.45	down
PGP(i-24:0/PGF1alpha)	0.38	-1.39	0.031	1.56	down
Allyl hexenoate	0.32	-1.64	0.022	1.61	down
5alpha-Androstane-3beta,7alpha,17beta-triol	0.23	-2.13	0.025	1.61	down
Saikosaponin H	0.34	-1.57	0.030	1.56	down
PGP(18:2(10E,12Z)+=O(9)/i-22:0)	0.27	-1.90	0.044	1.48	down
PI(22:2(13Z,16Z)/20:4(6E,8Z,11Z,13E)-2OH(5S,15S))	0.33	-1.59	0.031	1.57	down
CL(10:0/10:0/10:0/13:0)	0.47	-1.10	0.037	1.52	down
9(10)-EpOME	0.24	-2.06	0.015	1.74	down
9,10-DiHOME	0.41	-1.30	0.020	1.70	down
cis-trans-Nepetalactol	0.44	-1.18	0.024	1.67	down
2-trans,4-cis-Decadienoylcarnitine	0.37	-1.45	0.034	1.55	down
Notoginsenoside T2	0.34	-1.54	0.026	1.56	down
Erythromycylamine	0.29	-1.78	0.022	1.62	down
Taurodeoxycholic acid	0.33	-1.58	0.038	1.49	down
Chikusetsusaponin Ia	0.32	-1.65	0.027	1.60	down
PG(20:2(11Z,14Z)/18:2(9Z,12Z))	0.32	-1.64	0.032	1.57	down
PA(P-16:0/PGF2alpha)	0.22	-2.21	0.019	1.66	down
Pulchinenoside A	0.24	-2.06	0.021	1.67	down
PG(20:1(11Z)/20:3(6,8,11)-OH(5))	0.34	-1.57	0.035	1.56	down
PE(P-18:0/LTE4)	0.36	-1.49	0.036	1.53	down
Digoxigenin	0.26	-1.94	0.025	1.63	down
PC(P-18:1(11Z)/LTE4)	0.31	-1.67	0.028	1.61	down
PGP(PGF1alpha/i-24:0)	0.42	-1.25	0.031	1.57	down
PI(22:6(4Z,7Z,11E,13Z,15E,19Z)-2OH(10S,17)/20:0)	0.35	-1.51	0.028	1.60	down
16-Oxoandrostenediol	0.50	-1.01	0.031	1.51	down
2,3-Dinor-8-iso prostaglandin F2alpha	0.45	-1.15	0.027	1.56	down
11beta,21-Dihydroxy-5beta-pregnane-3,20-dione	0.37	-1.42	0.023	1.63	down
2-Hydroxy-4-[5-(4-methylphenyl)-3-(trifluoromethyl)pyrazol-1-yl]benzenesulfonamide	2.46	1.30	0.049	1.39	up
15-deoxy-delta12,14-Prostaglandin J2-2-glycerol ester	0.22	-2.17	0.023	1.64	down
PG(PGJ2/i-19:0)	0.37	-1.45	0.028	1.61	down
6,8a-Seco-6,8a-deoxy-5-oxoavermectin "1a" aglycone	3.48	1.80	0.020	1.72	up
PG(PGE1/i-19:0)	0.25	-1.98	0.021	1.66	down
PGP(i-13:0/a-25:0)	0.40	-1.32	0.029	1.58	down
PI(22:3(10Z,13Z,16Z)/5-iso PGF2VI)	0.28	-1.82	0.027	1.61	down
CL(10:0/11:0/11:0/11:0)	0.42	-1.26	0.031	1.56	down
PC(DiMe(13,5)/LTE4)	0.36	-1.48	0.030	1.57	down
(Z)-5-[(2R,3S,4S)-4-Hydroxy-2-[(E)-3-hydroxyoct-1-enyl]oxan-3-yl]pent-3-enoic acid	0.29	-1.80	0.031	1.57	down

trihydroxy-4-pregnen-3-one	0.27	-1.89	0.027	1.62	down
SM(d18:2(4E,14Z)/PGJ2)	0.33	-1.60	0.019	1.65	down
Ascorbyl palmitate	0.30	-1.76	0.022	1.65	down
PI(20:0/18:1(12Z)-2OH(9,10))	0.29	-1.78	0.014	1.72	down
Beloranib	0.42	-1.25	0.045	1.40	down
Benzquinamide	0.22	-2.17	0.021	1.54	down
PG(TXB2/20:1(11Z))	0.25	-2.01	0.029	1.52	down
LysoPE(0:0/18:2(9Z,12Z))	0.33	-1.59	0.038	1.45	down
1-Heneicosanoyl-glycero-3-phosphoserine	4.49	2.17	0.022	1.66	up
Permetin A	2.05	1.03	0.030	1.55	up
PS(PGJ2/18:0)	0.28	-1.83	0.031	1.45	down
PG(i-21:0/22:6(5Z,7Z,10Z,13Z,16Z,19Z)-OH(4))	0.37	-1.44	0.037	1.47	down
Penitrem D	2.49	1.32	0.040	1.56	up
PG(16:0/18:1(12Z)-O(9S,10R))	0.41	-1.29	0.032	1.48	down
PG(20:1(11Z)/22:6(4Z,8Z,10Z,13Z,16Z,19Z)-OH(7))	0.41	-1.30	0.040	1.48	down
Muzanzagenin	0.43	-1.20	0.043	1.44	down
Tenofovir exalidex	23.55	4.56	0.022	1.71	up
Ganodermic acid TQ	3.01	1.59	0.017	1.72	up
Ganglioside GA2 (d18:1/16:0)	4.27	2.09	0.024	1.70	up
N-Docosahexaenoyl Tyrosine	0.27	-1.89	0.027	1.51	down
Milbemycin A4	0.42	-1.26	0.026	1.52	down
Butyl oleate sulfate	0.31	-1.68	0.025	1.51	down
(R)-3-Hydroxy-hexadecanoic acid	0.45	-1.15	0.011	1.73	down
PE(18:0/22:2(13Z,16Z))	0.40	-1.33	0.024	1.64	down
4-Hydroxynonenol	2.17	1.12	0.022	1.69	up
3-Oxooctadecanoic acid	2.08	1.06	0.018	1.71	up
hydroxynonenal	2.11	1.08	0.021	1.69	up
Androsterone	0.33	-1.58	0.046	1.42	down
SM(d16:1/17:0)	0.34	-1.57	0.006	1.75	down
PC(LTE4/24:0)	0.23	-2.10	0.026	1.55	down
26-Hydroxyecdysone	0.23	-2.12	0.025	1.57	down
24,25-Dihydroxyvitamin D	0.41	-1.29	0.033	1.43	down
DG(13:0/PGF1alpha/0:0)	0.31	-1.68	0.025	1.61	down
(Z)-9-Cycloheptadecen-1-one	0.45	-1.16	0.019	1.65	down
13'-Hydroxy-gamma-tocotrienol	0.39	-1.35	0.011	1.69	down
Panatellin	0.40	-1.32	0.015	1.68	down
DG(14:0/18:3(6Z,9Z,12Z)/0:0)	0.46	-1.14	0.003	1.88	down
Disopyramide	0.41	-1.29	0.037	1.51	down
2,22-Dideoxy-3-dehydroecdysone	0.23	-2.13	0.029	1.49	down
3-Hydroxydodeca-5,7-dienoylcarnitine	0.48	-1.05	0.043	1.43	down
Cochliobolin A	0.46	-1.12	0.026	1.50	down
LysoPC(20:4(5Z,8Z,11Z,14Z)/0:0)	0.22	-2.21	0.019	1.64	down
PA(P-16:0/18:1(12Z)-2OH(9,10))	0.42	-1.24	0.049	1.43	down

Ganoderic acid U	0.40	-1.34	0.030	1.56	down
3-Oxo-12,18-ursadien-28-oic acid	0.46	-1.13	0.018	1.62	down
Tilmicosin	4.03	2.01	0.029	1.60	up
Ecdysone	0.41	-1.27	0.023	1.57	down
Basilol	0.42	-1.27	0.006	1.71	down
Ginsenoside Rh3	0.41	-1.29	0.017	1.67	down
Erythromycin	0.40	-1.33	0.003	1.74	down
1,25-Dihydroxy-19-norvitamin D3	0.34	-1.56	0.003	1.82	down
16alpha-Hydroxydehydroepiandrosterone	0.29	-1.79	0.041	1.53	down
Hovenidulcigenin A	0.26	-1.97	0.030	1.52	down
PGP(i-19:0/18:1(12Z)-O(9S,10R))	0.20	-2.30	0.024	1.53	down
Cortol	0.37	-1.43	0.009	1.74	down
PG(i-19:0/18:1(12Z)-2OH(9,10))	0.47	-1.09	0.037	1.51	down
DG(15:0/22:6(4Z,7Z,10Z,13E,15E,19Z)-OH(17)/0:0)	0.41	-1.28	0.018	1.70	down
18-Hydroxyoctadecadienoic acid	0.48	-1.06	0.049	1.41	down
(3S,3'R,4xi)-beta,beta-Carotene-3,3',4-triol	0.31	-1.67	0.009	1.66	down
Hydroxyspheroidene	0.39	-1.37	0.008	1.76	down
Maslinic Acid	0.48	-1.05	0.003	1.85	down
Karpoxanthin	0.24	-2.08	0.022	1.58	down
Calcidiol	0.42	-1.25	0.038	1.47	down
Polyoxyethylene sorbitan monooleate	0.32	-1.64	0.014	1.70	down
Prostaglandin J2	0.25	-1.99	0.030	1.59	down
PA(15:0/18:1(12Z)-2OH(9,10))	0.28	-1.84	0.017	1.70	down
Azitromycin	0.20	-2.29	0.015	1.70	down
Talinolol	0.40	-1.34	0.024	1.67	down
Nisoldipine	0.30	-1.74	0.049	1.35	down
5-Acetyl-2,3-dihydro-6,7-dimethyl-1H-pyrrolizine	0.40	-1.32	0.009	1.74	down
(10E,12E,14E)-9-Hydroxy-16-oxooctadeca-10,12,14-trienoylcarnitine	0.44	-1.19	0.007	1.76	down
(Z)-1-Methyl-2-(tridec-8-en-1-yl)quinolin-4(1H)-one	2.10	1.07	0.011	1.78	up
Ethyl cellulose	0.25	-1.98	0.027	1.49	down
(R)-Shinanolone	0.40	-1.33	0.008	1.76	down
3-Pyrrolidinecarboxylic acid, 4-(1,3-benzodioxol-5-yl)-1-(2-((2,6-diethylphenyl)amino)-2-oxoethyl)-2-(4-propoxyphenyl)-, (2R,3R,4S)-	0.25	-1.98	0.027	1.54	down
apicidin	0.12	-3.07	0.003	1.88	down
Cycloheximide	2.08	1.06	0.044	1.44	up
3-Methylglutarylcarnitine	2.07	1.05	0.034	1.54	up
all-trans-Hexaprenyl diphosphate	0.28	-1.84	0.020	1.62	down
Gluten exorphin C	0.15	-2.69	0.001	1.95	down
(5E)-3-Hydroxyhept-5-enoylcarnitine	0.41	-1.27	0.022	1.60	down
Dihydroethidium	0.30	-1.73	0.024	1.60	down

Koenigine	0.24	-2.05	0.008	1.75	down
Lipid X	0.18	-2.45	0.001	1.91	down
1-Formylneogrifolin	2.12	1.09	0.035	1.41	up
Farnesylcysteine	2.86	1.52	0.013	1.72	up
DG(16:1(9Z)/22:6(4Z,7Z,10Z,13Z,16Z,19Z)/0:0)	4.07	2.02	0.021	1.59	up
(10S)-Juvenile hormone III diol	0.34	-1.55	0.007	1.70	down
Propanidid	0.30	-1.72	0.018	1.65	down
(R)-2-Methylimino-1-phenylpropan-1-ol	0.28	-1.86	0.009	1.76	down
PE(14:1(9Z)/20:5(7Z,9Z,11E,13E,17Z)-3OH(5,6,15))	0.16	-2.66	0.001	1.94	down
2-O-Methyl-L-fucose	0.27	-1.86	0.046	1.47	down
Vephylline	0.50	-1.00	0.011	1.65	down
Hexadecyl ferulate	0.20	-2.31	0.018	1.53	down
N-Methylrosmaricine	0.38	-1.41	0.030	1.56	down
Piperochromanoic acid	0.38	-1.38	0.044	1.47	down
L-Arginine	0.39	-1.38	0.011	1.62	down
1-(2-(4-(3-Phenyl-2H-1-benzopyran-2-yl)phenoxy)ethyl)piperidine	0.42	-1.25	0.011	1.61	down
1-Hydroxyepiacorone	0.22	-2.21	0.020	1.54	down
PGP(i-20:0/LTE4)	0.21	-2.23	0.028	1.44	down
L-alpha-Amino-1H-pyrrole-1-hexanoic acid	0.40	-1.33	0.012	1.61	down
2-Propenamide, 2-cyano-3-(4-hydroxy-3,5-bis(1-methylethyl)phenyl)-	0.18	-2.50	0.006	1.71	down
Cofaryloside	0.18	-2.51	0.010	1.63	down
Neomycin B	0.13	-2.98	0.001	1.89	down
Testosterone	2.06	1.05	0.019	1.52	up
Pentahomomethionine	2.42	1.28	0.028	1.54	up
Pyrohyperforin	0.39	-1.35	0.040	1.53	down
7alpha-hydroxy-3-oxochol-4-en-24-oic Acid	2.05	1.04	0.014	1.63	up
LysoPA(0:0/18:1(9Z))	0.22	-2.21	0.016	1.64	down
Quillajasaponin	2.21	1.15	0.013	1.63	up
Glutathionylspermidine	0.21	-2.23	0.016	1.69	down
Gentamicin X2	0.22	-2.19	0.021	1.66	down
(5Z)-(15S)-11-alpha-hydroxy-9,15-dioxoprosta-13-enoate	0.31	-1.70	0.012	1.72	down
2-(4,4-Difluoro-1-piperidinyl)-6-methoxy-N-[1-(1-methylethyl)-4-piperidinyl]-7-[3-(1-pyrrolidinyl)propoxy]-4-quinazolinamine	5.80	2.54	0.020	1.71	up
Tetraglyme	0.24	-2.08	0.029	1.56	down
Flurithromycin	0.40	-1.32	0.044	1.50	down
24,25-Diacetylvulgaroside	0.36	-1.47	0.020	1.69	down
Polypodoside B	0.36	-1.48	0.012	1.72	down
Gentamicin B	0.42	-1.25	0.029	1.58	down
Heptaethylene glycol monododecyl ether	3.90	1.96	0.021	1.69	up

1-Isopropyl-N-((6-methyl-2-oxo-4-propyl-1,2-dihydropyridin-3-yl)methyl)-6-(2-(4-methylpiperazin-1-yl)pyridin-4-yl)-1H-indazole-4-carboxamide	0.41	-1.29	0.049	1.46	down
Prizidilol	71327598.41	26.09	0.020	1.70	up
Bis(adenosine)-5'-pentaphosphate	4.33	2.11	0.008	1.82	up
Linalyl anthranilate	2.45	1.29	0.011	1.80	up
17beta-Estradiol-3,4-quinone	12.53	3.65	0.014	1.76	up
Dynorphin B (6-9)	13.53	3.76	0.021	1.69	up
Tryptophyl-Valine	209951819.45	27.65	0.019	1.72	up
DG(10:0/PGE2/0:0)	4.56	2.19	0.009	1.83	up
Heteroxanthin	104.98	6.71	0.018	1.74	up
(-)-11-Hydroxy-9,15,16-trioxooctadecanoic acid	3.56	1.83	0.010	1.68	up
PA(14:0/PGJ2)	770519019.84	29.52	0.022	1.70	up
Bacoside A	755603835.96	29.49	0.021	1.72	up
Torvoside E	0.24	-2.03	0.048	1.35	down
PI(16:1(9Z)/18:3(10,12,15)-OH(9))	0.26	-1.97	0.049	1.35	down
(2R,3S)-3-(6-Amino-9H-purin-9-yl)nonan-2-ol	0.28	-1.84	0.014	1.65	down
Isoleucyl-Lysine	0.15	-2.76	0.003	1.92	down
Avermectin B1b aglycone	0.42	-1.25	0.027	1.52	down
Geranic acid	0.39	-1.37	0.002	1.95	down
Milbemycin D	0.43	-1.20	0.022	1.67	down
Deoxycholylarginine	0.34	-1.57	0.030	1.55	down
12(13)-epoxy-6Z,9Z-octadecadienoic acid	0.33	-1.59	0.011	1.75	down
PS(20:0/PGF1alpha)	0.27	-1.90	0.013	1.76	down
PS(22:1(13Z)/PGF1alpha)	0.27	-1.87	0.013	1.77	down
Peregrine	0.31	-1.71	0.010	1.80	down
PI(PGE2/22:2(13Z,16Z))	0.48	-1.07	0.015	1.70	down
LysoPE(0:0/24:6(6Z,9Z,12Z,15Z,18Z,21Z))	0.22	-2.19	0.013	1.71	down
DG(2:0/20:3(8Z,11Z,14Z)-2OH(5,6)/0:0)	0.34	-1.58	0.005	1.81	down
Scyphostatin	0.34	-1.55	0.010	1.79	down
Antibiotic JI-20A	0.38	-1.40	0.007	1.76	down
9,10,13-Trihydroxystearic acid	0.18	-2.50	0.003	1.92	down
PG(22:4(7Z,10Z,13Z,16Z)/22:6(5Z,7Z,10Z,13Z,16Z,19Z)-OH(4))	0.37	-1.43	0.011	1.79	down
PA(20:0/20:5(7Z,9Z,11E,13E,17Z)-3OH(5,6,15))	0.33	-1.61	0.011	1.79	down
Punicic acid	0.31	-1.70	0.010	1.80	down
PA(22:2(13Z,16Z)/6 keto-PGF1alpha)	0.33	-1.62	0.011	1.79	down
2(R)-HOT	0.30	-1.72	0.017	1.73	down
Ganglioside GM3 (d18:1/20:0)	17.97	4.17	0.019	1.73	up
PA(24:0/LTE4)	0.44	-1.17	0.017	1.73	down
12,13-DHOME	0.29	-1.76	0.004	1.92	down
9,10-DHOME	0.35	-1.53	0.009	1.83	down
PG(20:2(11Z,14Z)/PGJ2)	0.30	-1.73	0.007	1.88	down

PG(20:2(11Z,14Z)/20:4(5Z,8Z,11Z,14Z)-OH(20))	0.28	-1.86	0.009	1.84	down
[(2R)-3-[[(2R,3S,4S,5R)-5-(4-Amino-2-oxopyrimidin-1-yl)-4-cyano-3-hydroxyoxolan-2-yl]methoxy-hydroxyphosphoryl]oxy-2-hexadecanoyloxypropyl] hexadecanoate	0.29	-1.79	0.010	1.82	down
PC(20:1(11Z)/LTE4)	0.37	-1.42	0.008	1.82	down
[3h]Thienylcyclohexylpiperidine	0.16	-2.61	0.006	1.86	down
12-Hydroxy-8,10-octadecadienoic acid	0.29	-1.77	0.009	1.83	down
PE(P-18:0/22:6(5Z,8E,10Z,13Z,15E,19Z)-2OH(7S,17S))	0.35	-1.50	0.009	1.82	down
PE(20:1(11Z)/22:6(5Z,7Z,10Z,13Z,16Z,19Z)-OH(4))	0.29	-1.78	0.010	1.81	down
Mianserin	0.32	-1.63	0.046	1.51	down
Gentamicin C2	0.31	-1.71	0.007	1.84	down
Dactimicin	0.33	-1.62	0.009	1.80	down
PI(6 keto-PGF1alpha/22:2(13Z,16Z))	10.09	3.33	0.013	1.77	up
PE(20:2(11Z,14Z)/20:5(5Z,8Z,11Z,14Z,17Z))	0.32	-1.62	0.008	1.84	down
Colistin A	19.47	4.28	0.017	1.75	up
Octyl 2-furoate	0.14	-2.86	0.002	1.94	down
SM(d18:0/20:5(7Z,9Z,11E,13E,17Z)-3OH(5,6,15))	0.30	-1.74	0.008	1.83	down
PS(22:0/18:1(12Z)-2OH(9,10))	0.29	-1.80	0.009	1.83	down
PE(22:4(7Z,10Z,13Z,16Z)/PGE1)	0.31	-1.68	0.007	1.84	down
PS(DiMe(13,5)/MonoMe(13,5))	0.36	-1.47	0.007	1.84	down
(6R,8Z)-6-Hydroxy-3-oxotetradecenoic acid	0.27	-1.91	0.001	1.88	down
PC(22:3(10Z,13Z,16Z)/6 keto-PGF1alpha)	0.33	-1.60	0.008	1.82	down
Dodecyl-beta-D-maltoside	0.48	-1.05	0.013	1.73	down
Muroctasin	0.34	-1.54	0.013	1.77	down
Amino (2S)-2-amino-4-methylpentanoate	0.30	-1.72	0.008	1.68	down
20-HEDE	0.31	-1.67	0.004	1.78	down
9-Hydroxyoctadeca-2,4-dienoic acid	0.43	-1.21	0.044	1.43	down
1,1,1-Trifluorohenicosa-6,9,12,15-tetraene	0.29	-1.79	0.013	1.62	down
PS(24:0/20:4(6E,8Z,11Z,14Z)+=O(5))	0.36	-1.49	0.011	1.74	down
Elastin	2.63	1.39	0.029	1.62	up
Triethylene glycol monohexyl ether	0.27	-1.91	0.049	1.48	down
10-Deoxymethymycin	0.17	-2.54	0.007	1.67	down
(4Z,7Z,10E,12E,16Z)-18-(3-Ethylcycloprop-1-en-1-yl)-14-hydroxyoctadeca-4,7,10,12,16-pentaenoylcarnitine	0.33	-1.60	0.027	1.49	down
Surfactin A	0.31	-1.71	0.026	1.52	down
DG(10:0/5-iso PGF2VI/0:0)	3.48	1.80	0.044	1.53	up
Collettiside I	4.26	2.09	0.024	1.67	up
PA(i-12:0/18:2(9Z,11Z))	3.59	1.84	0.033	1.58	up
SM(d18:1/PGJ2)	0.20	-2.36	0.006	1.87	down
20,26-Dihydroxyecdysone	3.27	1.71	0.013	1.73	up

Surfactin	0.47	-1.09	0.010	1.66	down
PS(MonoMe(13,5)/MonoMe(13,5))	0.45	-1.15	0.011	1.70	down
Palmitic acid	0.44	-1.18	0.017	1.60	down
UDP-2-acetamido-4-(D-alanylamino)-2,4,6-trideoxy-alpha-D-glucose	5.86	2.55	0.025	1.64	up
gamma-Eudesmol rhamnoside	0.29	-1.77	0.001	1.89	down
3-O-beta-D-glucosyl-brassicasterol	0.34	-1.56	0.002	1.82	down
Milbemycin beta1	0.41	-1.27	0.042	1.51	down
Mepindolol	0.27	-1.91	0.013	1.63	down
PS(6 keto-PGF1alpha/22:0)	0.39	-1.36	0.027	1.63	down
Jervine	0.37	-1.44	0.044	1.52	down
Capsicoside A1	0.36	-1.45	0.025	1.50	down
Polymyxin M	3.46	1.79	0.024	1.64	up
Procaine	0.18	-2.44	0.010	1.79	down
LysoPE(15:0/0:0)	0.31	-1.71	0.007	1.86	down
Ganglioside GM3 (d18:1/18:1(11Z))	2.45	1.29	0.044	1.55	up
Ganglioside GM3 (d18:1/9Z-18:1)	9.42	3.24	0.033	1.62	up
PS(24:0/18:1(12Z)-O(9S,10R))	0.49	-1.02	0.022	1.63	down
PC(DiMe(11,5)/22:6(4Z,7Z,11E,13Z,15E,19Z)-2OH(10S,17))	0.41	-1.27	0.025	1.60	down
PG(i-19:0/PGF1alpha)	0.36	-1.46	0.026	1.64	down
PE(P-18:0/PGJ2)	0.40	-1.31	0.008	1.85	down
Azithromycin	0.40	-1.32	0.005	1.84	down
Neoxanthin	0.30	-1.76	0.021	1.68	down
(3b,6b,8a,12a)-8,12-Epoxy-7(11)-eremophilene-6,8,12-trimethoxy-3-ol	861243367.76	29.68	0.020	1.70	up
PG(i-24:0/20:3(8Z,11Z,14Z)-2OH(5,6))	0.36	-1.47	0.004	1.92	down
Rumenic acid	0.39	-1.37	0.017	1.62	down
Acetyl-pepstatin	0.32	-1.64	0.016	1.75	down
20-Deoxynarasin	0.48	-1.05	0.005	1.78	down
Dihydromycoplanecin A	24.32	4.60	0.021	1.69	up
(2S)-6-Hydroxy-2,7,8-trimethyl-2-[(4R,8R)-4,8,12-trimethyltridecanoyl]-3,4-dihydrochromene-5-carbaldehyde	0.48	-1.07	0.002	1.82	down
CL(8:0/10:0/18:0/18:2(9Z,11Z))	0.34	-1.54	0.003	1.83	down
PS(22:2(13Z,16Z)/22:5(7Z,10Z,13Z,16Z,19Z))	0.47	-1.10	0.005	1.82	down
Glabracin A	18.32	4.20	0.017	1.75	up
PA(a-25:0/PGF2alpha)	0.33	-1.60	0.007	1.85	down
(2S,3S,5S,8R,9S,10S,13S,14S,16S,17R)-17-Acetyloxy-10,13-dimethyl-2-morpholin-4-yl-16-(1-prop-2-enylpyrrolidin-1-ium-1-yl)-2,3,4,5,6,7,8,9,11,12,14,15,16,17-tetradecahydro-1H-cyclopenta[a]phenanthren-3-olate	0.28	-1.84	0.004	1.91	down
Hovenidulcigenin B	0.17	-2.54	0.007	1.86	down

LysoPC(18:2(9Z,12Z)/0:0)	0.29	-1.78	0.002	1.86	down
Glycocholate	0.13	-2.91	0.002	1.96	down
Ponasterone A	0.36	-1.47	0.006	1.84	down
SM(d20:1/PGF2alpha)	0.30	-1.75	0.008	1.84	down
Trihexosylceramide (d18:1/26:1(17Z))	13.88	3.79	0.020	1.70	up
(±)-(E)-3-Methyl-4-decen-1-yl acetate	0.05	-4.22	0.000	2.04	down
3 alpha,7 alpha,26-Trihydroxy-5beta-cholestane	0.26	-1.93	0.011	1.70	down
PG(i-22:0/PGJ2)	0.32	-1.66	0.007	1.83	down
PG(20:1(11Z)/20:4(5Z,8Z,11Z,14Z)-OH(20))	0.27	-1.87	0.007	1.83	down
PG(PGF1alpha/20:1(11Z))	0.31	-1.71	0.010	1.83	down
PA(21:0/PGJ2)	0.34	-1.55	0.006	1.86	down
Pheophytin a	0.37	-1.43	0.007	1.81	down
PS(18:0/18:2(9Z,12Z))	0.41	-1.29	0.001	1.91	down
4alpha-Methylzymosterol-4-carboxylate	0.14	-2.83	0.002	1.90	down
sphingosylphosphorylcholine	0.16	-2.69	0.003	1.85	down
DG(8:0/0:0/17:0)	0.11	-3.20	0.000	1.97	down
Valnemulin	9.99	3.32	0.020	1.71	up
solasodine 3-O-beta-D-glucopyranoside	608079826.51	29.18	0.020	1.75	up
PC(24:0/LTE4)	5.94	2.57	0.019	1.72	up
SM(d17:2(4E,8Z)/PGF2alpha)	0.30	-1.75	0.008	1.84	down
oleandomycin	4.04	2.02	0.020	1.71	up
1,2-Didecanoyl PC	0.18	-2.51	0.006	1.83	down
DG(18:0/PGJ2/0:0)	2.27	1.18	0.025	1.66	up
Ibogamine	0.14	-2.81	0.002	1.91	down
3-O-Mycarosylerythronolide B	0.14	-2.82	0.002	1.94	down
SM(d20:1/TXB2)	0.22	-2.20	0.012	1.78	down
SM(d18:2(4E,14Z)/PGF1alpha)	0.26	-1.92	0.011	1.79	down
DG(8:0/PGE1/0:0)	64.60	6.01	0.017	1.73	up
Cuscohygrine	0.20	-2.35	0.002	1.82	down
Hexosylsphingosine	0.07	-3.80	0.000	2.03	down
(5Z)-7-[(1R,2R,5S)-5-Hydroxy-2-[(1E,3S,5Z)-3-hydroxyocta-1,5-dien-1-yl]-3-oxocyclopentyl]hept-5-enoylcarnitine	0.14	-2.87	0.002	1.83	down
SM(d17:2(4E,8Z)/5-iso PGF2VI)	0.30	-1.73	0.006	1.86	down
PE(24:1(15Z)/LTE4)	3.06	1.61	0.019	1.71	up
(1R,3S,5Z)-5-[(2Z)-2-[(1R,3Ar,7aS)-1-[(2S)-1-[3-(2-hydroxypropan-2-yl)phenoxy]propan-2-yl]-7a-methyl-2,3,3a,5,6,7-hexahydro-1H-inden-4-ylidene]ethylidene]-4-methylidenecyclohexane-1,3-diol	2.21	1.14	0.019	1.70	up
N-Arachidonoyl Glutamic acid	0.35	-1.52	0.006	1.86	down
Retinyl palmitate	0.03	-5.24	0.003	1.96	down
PG(16:0/PGF1alpha)	0.30	-1.73	0.006	1.85	down
1-Stearoyl-2-hydroxy-sn-glycero-3-phosphocholine	0.02	-5.40	0.001	2.01	down

PS(20:0/18:1(12Z)-O(9S,10R))	0.42	-1.27	0.005	1.86	down
Isobutyl 10-undecenoate	0.04	-4.70	0.001	1.99	down
PE-NMe(14:1(9Z)/20:1(11Z))	0.39	-1.36	0.006	1.87	down
PG(20:3(5Z,8Z,11Z)/18:1(12Z)-2OH(9,10))	0.44	-1.18	0.005	1.83	down
PC(22:0/6 keto-PGF1alpha)	0.18	-2.45	0.004	1.92	down
PE-NMe(20:2(11Z,14Z)/20:5(5Z,8Z,11Z,14Z,17Z))	0.38	-1.39	0.004	1.85	down
PC(18:0/PGF1alpha)	0.16	-2.63	0.003	1.94	down
Ricinoleic Acid methyl ester	6.95	2.80	0.043	1.50	up
PE(14:1(9Z)/22:4(7Z,10Z,13Z,16Z))	0.41	-1.29	0.002	1.90	down
Asparagoside A	2.84	1.50	0.047	1.49	up
Corepoxylone	3.70	1.89	0.045	1.51	up
Citronellyl hexanoate	3.23	1.69	0.021	1.70	up
PE(15:0/18:1(11Z))	0.23	-2.14	0.002	1.98	down
Bornaprolol	0.32	-1.65	0.001	1.99	down
9S,10R-Epoxy-6Z-octadecene	4.79	2.26	0.042	1.53	up
(+)-15,16-Dihydroxyoctadecanoic acid	0.16	-2.66	0.004	1.86	down
Lasinavir	4.10	2.03	0.049	1.49	up
PS(24:0/PGF2alpha)	0.30	-1.74	0.002	1.88	down
PS(22:0/20:3(8Z,11Z,14Z)-2OH(5,6))	0.25	-1.99	0.001	1.90	down
35S-Methylokadaic acid 7-hexadecanoate	2.17	1.11	0.042	1.51	up
PS(20:2(11Z,14Z)/20:2(11Z,14Z))	0.26	-1.96	0.001	1.90	down
PG(i-19:0/PGE1)	0.27	-1.89	0.003	1.88	down
PS(20:2(11Z,14Z)/PGF1alpha)	0.48	-1.07	0.008	1.73	down
N-di-Demethyl roxithromycin	0.30	-1.71	0.003	1.90	down
Prolyl-phenylalanyl-N-heptylargininamide	2.36	1.24	0.032	1.58	up
PA(a-25:0/PGJ2)	0.46	-1.11	0.003	1.86	down
SM(d20:1/PGJ2)	0.40	-1.30	0.002	1.89	down
PE-NMe(18:1(11Z)/18:2(9Z,12Z))	0.48	-1.07	0.009	1.83	down
PC(P-16:0/20:4(8Z,11Z,14Z,17Z)-2OH(5S,6R))	0.35	-1.53	0.002	1.90	down
DG(LTE4/16:0/0:0)	2.08	1.06	0.031	1.61	up
PS(24:0/20:5(5Z,8Z,11Z,14Z,16E)-OH(18R))	2.06	1.04	0.022	1.56	up
12S-HHT	0.18	-2.50	0.001	1.98	down
PE(17:2(9Z,12Z)/14:1(9Z))	2.41	1.27	0.022	1.68	up
Furazabol	2.50	1.32	0.017	1.69	up
N-Myristoyl Threonine	2.89	1.53	0.020	1.72	up
PE-NMe(14:0/18:0)	0.34	-1.56	0.010	1.72	down
2-Hydroxydodecyl methacrylate	0.50	-1.01	0.003	1.87	down
Ciprostene	11.15	3.48	0.018	1.74	up
(1R,4E,5'R,6R,6'R,7S,8S,10S,11S,12R,14S,15R,16S,18E,20E,22S,25R,27S,29S)-22-Ethyl-7,11,14,15-tetrahydroxy-6'-(2-hydroxypropyl)-5',6,8,10,12,14,16,29-octamethylspiro[2,26-dioxabicyclo[23.3.1]nonacosa-4,18,20-triene-27,2'-oxane]-3,9,13-trione	0.34	-1.56	0.001	1.92	down

Frangulanine	0.37	-1.44	0.001	1.93	down
LysoPA(24:0/0:0)	0.41	-1.30	0.005	1.88	down
Quinquenoside F1	0.40	-1.32	0.002	1.89	down
LysoPC(P-16:0/0:0)	0.32	-1.65	0.006	1.85	down
N-Myristoyl Asparagine	9.40	3.23	0.017	1.74	up
Dolicholide	0.26	-1.94	0.008	1.85	down
3-Dehydroteasterone	0.23	-2.10	0.012	1.79	down
Palmitoyl Ara-C	0.27	-1.91	0.005	1.85	down
PS(14:0/18:2(9Z,12Z))	3.70	1.89	0.019	1.72	up
PGP(i-24:0/PGE1)	0.28	-1.81	0.002	1.92	down
N-Docosahexaenoyl Glutamine	0.24	-2.07	0.007	1.86	down
12-Hydroxy-12-octadecanoylcarnitine	0.13	-2.96	0.001	1.93	down
1-Heptadecanoylglycerophosphoethanolamine	0.17	-2.54	0.007	1.85	down
PG(i-12:0/i-18:0)	2.88	1.53	0.023	1.67	up
Polysorbate 60	0.21	-2.25	0.007	1.86	down
DG(13:0/22:6(5Z,8E,10Z,13Z,15E,19Z)-2OH(7S, 17S)/0:0)	0.40	-1.34	0.000	2.01	down
13-Demethylspirolide C	0.32	-1.65	0.001	1.93	down
Glycerol 1-(5-hydroxydodecanoate)	0.50	-1.00	0.003	1.84	down
3-O-Sulfogalactosylceramide (d18:1/24:0)	0.49	-1.02	0.003	1.94	down
4-Hydroxy-3-(16-methylheptadecyl)-2H-pyran-2-one	0.38	-1.39	0.004	1.92	down
PS(20:0/20:0)	0.48	-1.07	0.003	1.96	down
Cer(d18:0/LTE4)	0.38	-1.40	0.002	1.97	down
PC(P-18:0/18:1(12Z)-2OH(9,10))	0.48	-1.05	0.002	1.98	down
DG(17:0/6 keto-PGF1alpha/0:0)	0.46	-1.13	0.002	1.96	down
3a,21-Dihydroxy-5b-pregnane-11,20-dione	2.95	1.56	0.030	1.58	up
PE(15:0/18:3(9Z,12Z,15Z))	2.63	1.39	0.016	1.74	up
PE(16:0/18:3(9Z,12Z,15Z))	2.34	1.23	0.021	1.73	up
Cer(d18:0/PGJ2)	2.08	1.06	0.014	1.74	up
Metoprolol	0.44	-1.20	0.020	1.71	down
Homodolicholide	0.49	-1.02	0.008	1.83	down
11-Keto-.beta.-boswellic acid	0.41	-1.30	0.011	1.80	down
(R)-14-Methyloxacyclotetradecan-2-one	0.20	-2.32	0.015	1.75	down
Ganolucidic acid E	0.44	-1.20	0.007	1.84	down
PG(i-24:0/20:4(6E,8Z,11Z,14Z)+=O(5))	0.31	-1.69	0.013	1.79	down
Triton X-100	0.26	-1.94	0.005	1.77	down
Annomuricin A	58.31	5.87	0.018	1.76	up
PA(24:0/PGE2)	0.38	-1.41	0.017	1.73	down
SM(d19:1/PGE2)	0.37	-1.43	0.020	1.71	down
DG(19:0/6 keto-PGF1alpha/0:0)	0.37	-1.45	0.018	1.72	down
PE(15:0/P-18:0)	0.39	-1.37	0.024	1.65	down
iso-A2E(11-cis)	0.48	-1.06	0.048	1.47	down
Oleic Acid ethyl ester	0.44	-1.19	0.043	1.47	down

Ganglioside GA2 (d18:1/20:0)	2.21	1.14	0.039	1.43	up
